# Characterization of HIV-1 entry inhibitors with broad activity against R5 and X4 viral strains

**DOI:** 10.1186/s12967-015-0461-9

**Published:** 2015-04-02

**Authors:** Francesca Sironi, Mauro Malnati, Nicola Mongelli, Paolo Cozzi, Christina Guzzo, Silvia Ghezzi, Carles Martínez-Romero, Adolfo García-Sastre, Paolo Lusso, Daniela Jabes, Priscilla Biswas

**Affiliations:** Unit of Human Virology, Division of Immunology, Transplantation and Infectious Diseases, San Raffaele Hospital, Via Olgettina 60, 20132 Milan, Italy; Unit of Viral Pathogens and Biosafety, Division of Immunology, Transplantation and Infectious Diseases, San Raffaele Hospital, Via Olgettina 60, 20132 Milan, Italy; Via Tertulliano 38, 20137 Milano, Italy; Via Zanella 48/5, Milano, 20133 Italy; Laboratory of Immunoregulation, National Institute of Allergy and Infectious Diseases (NIAID), National Institutes of Health (NIH), Bethesda, MD 20892 USA; Department of Microbiology, New York, NY 10029 USA; Global Health and Emerging Pathogens Institute, New York, NY 10029 USA; Department of Medicine, Division of Infectious Diseases, Icahn School of Medicine at Mount Sinai, New York, NY 10029 USA; NeED Pharmaceuticals srl, Viale Ortles 22/4, 20139 Milan, Italy; Unit of Molecular Immunology, Division of Genetics and Cell Biology, San Raffaele Hospital, Via Olgettina 60, 20132 Milan, Italy

## Abstract

**Background:**

Combined antiretroviral therapy has drastically reduced mortality and morbidity of HIV-infected individuals. Nevertheless long-term toxicity and appearance of viral resistance hampers the prolonged effectiveness of combination therapy, requiring a continuous input of drugs to replace those utilized in combination regimens. We here investigated the anti-HIV activity of novel derivatives of the suradista chemical class.

**Methods:**

Compounds were tested on acute HIV-1 infection of activated peripheral blood mononuclear cells. HIV production was monitored by enzyme-linked immunosorbent assay measuring the protein p24 released in culture supernatants. Fusion assays were carried out to study the mechanism of action of these compounds. A modified version of a previously established recombinant vaccinia virus-based assay was used measuring activation of a reporter gene upon fusion of two distinct cell populations. Flow cytometry was performed in competition assays for the binding of several antibodies targeting different sites of the viral envelope glycoprotein gp120, or the receptor CD4, or the coreceptors CXCR4 and CCR5.

**Results:**

Four compounds inhibited replication of a prototypic R5 (BaL) and X4 (IIIB) laboratory-adapted HIV-1 strain at low micromolar concentrations, in the absence of cytotoxicity. Approximately a ten fold greater activity was achieved against the X4 as compared to the R5 strain.

The compounds blocked X4 and R5 HIV-1 fusion, a step of viral entry. This activity appeared specific for HIV-1, as entry of human herpesvirus 6 (HHV-6) and influenza virus was not substantially affected. Further investigation of the inhibitory mechanism revealed that these new molecules target the viral envelope, rather than the coreceptors, as previously shown for a congener of the same class characterized by a long plasmatic half-life. Indeed ND-4043, the most active compound, specifically competed with binding of monoclonal antibodies against the CD4-binding site (CD4-BS) and coreceptor-binding site (CoR-BS) of gp120. These compounds displayed broad anti-HIV activity, as they inhibited various primary R5, X4 and, importantly, dualtropic R5X4 HIV-1 isolates. Of the four derivatives tested, the dimeric compounds were consistently more potent than the monomeric ones.

**Conclusions:**

Given their unique features, these molecules represent promising candidates for further development and exploitation as anti-HIV therapeutics.

**Electronic supplementary material:**

The online version of this article (doi:10.1186/s12967-015-0461-9) contains supplementary material, which is available to authorized users.

## Background

Despite the success of global treatment and prevention strategies, HIV infection rates are still growing worldwide, and AIDS remains a significant public health burden in low- to middle-income countries. Combination antiretroviral therapy (ART), encompassing a cocktail of drugs targeting different steps of the viral life cycle [1], is the standard treatment regimen, resulting in slowed disease progression and significantly prolonged life expectancy of patients. Indeed, current inhibitors include a wide array of viral targets, such as viral enzymes (reverse-transcriptase, protease, integrase), viral structural proteins (gp41), and host cellular components, such as the chemokine receptor CCR5, which is the major HIV-1 coreceptor, in addition to CXCR4.

Despite these advancements, mutations in HIV-1 can arise which confer resistance to drugs, often resulting in resistance to entire inhibitor classes. Moreover, long-term drug toxicity, although reduced in comparison to early drugs, remains a critical factor in determining the patient outcome and long-term health. Therefore, it is evident that clinical management of HIV requires novel drugs to be continuously available for inclusion in ART regimens.

Herein, we report the anti-HIV-1 activity of novel synthetic molecules and elucidate their mechanism of action. They belong to the suradista chemical class which shares certain features with the anti-trypanosoma drug suramin [2,3] and the antibiotic distamycin [4]. Suramin itself was shown early on to counteract the cytopathic effect of HIV *in vitro* [5], but in the following clinical trials it did not result as a clear benefit for AIDS patients [6,7]. Despite binding to the minor groove of DNA, most of the biological effects of distamycin were likely due to the interaction with membrane structures [8]. The anti-angiogenic activity of suradista molecules has been investigated *in vitro* [9] as well as in a clinical phase-I study for the treatment of cancer [10]. Several sulfonated and phosphonated suradista molecules have been evaluated as HIV inhibitors [11], and certain congeners have been shown to interact with HIV coreceptors [12]. We here demonstrate that novel suradista compounds act as HIV entry inhibitors targeting critical determinants of the viral envelope of both R5 and X4 HIV-1 viruses. This remarkable feature, along with the pharmacokinetic properties of members of the suradista family, warrants further investigation and development of these molecules.

## Methods

### Reagents

The experimental compounds herein tested were dissolved in DMSO at a stock concentration of 15 mM, aliquoted and frozen at –20°C. The aliquot in use was kept at room temperature (rt). Control cells always contained DMSO at a concentration corresponding to that of the highest concentration of the compounds.

Cells were cultured in complete medium consisting of RPMI 1640 or DMEM (the latter for fusion assays) (Euroclone) supplemented with antibiotics penicillin/streptomicin and glutamin plus 10% heat-inactivated fetal calf serum (FCS) (Euroclone).

Maraviroc and AMD3100 were obtained from the Centre for AIDS Reagents, National Institute for Biological Standards & Control (NIBSC), UK (referred to as NIBSC, UK).

### HIV-1 acute infections

Peripheral blood mononuclear cells (PBMC) were prepared by Ficoll density gradient (Lympholyte H) centrifugation of buffy coats from healthy donors. PBMC were routinely viable at 95-98% and activated with phytoemagglutinin (PHA) for 48h. Stock titered preparations of HIV-1 IIIB and BaL were used in adsorption (30min at 37°C) to infect PHA-activated PBMC, then excess virus was washed away. Infected cells were maintained in complete RPMI medium plus IL-2 (Proleukin, Novartis) at 200 U/ml. Compounds were added prior to adsorption and then readded in the 96-well culture plates seeded with PBMC in triplicate. Cell culture supernatant was harvested at day 4 and 7 post-infection and frozen at -20°C until tested for viral replication; compounds were replaced at day 4 along with fresh complete medium.

### Cytotoxicity assay

PBMC and TZM-bl viability was measured by the MTT [3-(4,5- dimethylthiazol-2-yl)-2,5-diphenyl tetrazolium bromide; Sigma] assay. Cells were seeded in 96-well plates in three replicates and were incubated for four (TZM-bl) or six (PBMC) days with different concentrations of the compounds or DMSO alone at 0.2% as control. Then 10μl of complete medium containing MTT (0.5 mg/ml) was added to each well. After 24h incubation at 37°C, the supernatant was removed and 200μl of ethanol was added to each well to solubilize the formazan crystals. After vigorous shaking, absorbance was measured in a microplate reader at 490 nm.

### Viral replication assays

The HIV-1 structural protein p24 Gag was measured by a twin-site sandwich enzyme-linked immunosorbent assay (ELISA) (Aalto Bio Reagents Ltd, Dublin, Ireland), based on a previously published method [13]. Briefly, p24 antigen is captured from a detergent lysate of virions present in culture supernatants by a sheep polyclonal antibody adsorbed to a solid phase (3h incubation at rt). Bound p24 is detected with a mouse alkaline phosphatase-conjugated anti-p24 moAb (1h incubation) and a luminescent detection system. The luminescence readout gives a broader dynamic range than the colorimetric readout and allows more accurate quantification of p24.

In preliminary experiments viral replication was also measured with the radioactive reverse-transcriptase activity assay (RT assay), as previously described [14].

### Cell lines

PM1 [15] (from Dr. P. Lusso, AIDS Research and Reference Program, Division of AIDS, NIAID, NIH, USA; hereafter referred to as NIH, USA) is a clonal derivative of the human T lymphocytic HUT 78 cell line characterized by a unique susceptibility to a wide range of HIV-1 isolates, including R5 strains. Sup T1 (NIH, USA, from Dr. J. Hoxie) is a non-Hodgkin T cell line [16]. MDCK (NIBSC, UK) are Madin-Darby normal canine kidney cells and HOS is a human osteosarcoma cell line (NIBSC, UK, from Dr. D. Littman and Dr. V Kewal Ramni).

### HIV-1 Fusion assays

The HIV-1 fusion assay is based on the vaccinia virus T7 RNA polymerase expression system with recombinant derivatives of the vaccinia virus (v) strain WR obtained through the NIH, USA [17]. “Effector” or “Donor” cells are infected with vTF7-3 expressing the bacteriophage T7 RNA polymerase gene under the control of the v p7.5 promoter from Dr. T. Fuerst and Dr B. Moss. “Target” or “Reporter” cells are infected with vCB21R-lacZ, expressing the E. coli lacZ gene linked to the bacteriophage T7 RNA polymerase promoter from Dr. C.C Broder, Dr P.E. Kennedy and Dr. E.A. Berger. In addition donor cells express HIV-1 envelope glycoproteins, whereas target cells also express CD4 plus CXCR4 or CCR5. When donor and target cells are co-cultured (2h at 37°C) and have the appropriate HIV entry molecules they fuse, allowing the T7 RNA polymerase to pass from the donor to the target cell, bind to its promoter and activate the lacZ gene. Subsequent production of β-galactosidase in the cytoplasm is assessed by a colorimetric assay of detergent cell lysates with optical density (OD) as the final read out. In this study the following donor cells have been utilized: murine embryo fibroblast 3T3 cells (NIH, USA) infected with vaccinia virus expressing X4 (vCB41) or R5 (vCB43) HIV-1 envelope, Sup-T1 persistently infected with HIV-1 IIIB (clade B, X4) and PM1 cells persistently infected with the following HIV-1 viruses: IIIB (clade B, X4), MN (clade B, X4), 92UG027 (clade D, X4), NP1525 (clade CRF01_AE, X4), B3-65 (clade B, X4), BaL (clade B, R5), SF162 (clade B, R5), 92BR025 (clade C, R5), 92RW009 (clade A, R5), CM244 (clade E, R5), 92US077 (clade B, R5X4) and HIV-2: 6669 (R5X4). Target cells used in this study were 3T3 cells stably transfected with CD4 plus CXCR4/CCR5 and the TZM-bl cell line (NIH, USA, from Dr. John C. Kappes, Dr. Xiaoyun Wu and Tranzyme Inc.). TZM-bl [18] derived from a clone of the HeLa (human cervix carcinoma) cell line that stably expresses large amounts of CD4 and CCR5, in addition to CXCR4.

Donor and target cells were cultured in DMEM (Euroclone) plus 2.5% of FCS and incubated for 2h in the absence or presence of ND-4043 prior to lysis with NP-40 0.5%, followed by addition of β-galactosidase substrate and reading absorbency of the plates at 570nm with an ELISA microplate reader (Biorad 680). In the “wash” experimental conditions the compound ND-4043 was incubated 30min at 37°C with either donor or target cells, washed away and not replaced in the co-culture. The β-galactosidase substrate chlorophenol red-beta-D-galactopyranoside (CPRG) was purchased from Roche Applied Sciences.

### Flow cytometry

Monoclonal antibodies (moAbs) directed against HIV-1 envelope, CCR5 (clone 2D7) and CXCR4 (clone 12G5, 44708, 44716, and 44717) were obtained from NIH, USA and NIBSC, UK. Indirect immunofluorescence was performed using phycoerythrin (PE)-conjugated goat anti-human and anti-mouse immunoglobulins (Southern Biotech). sCD4 (NIBSC, UK) at 5μg/ml was added to 3T3 cells prior to labelling with 17b and 48d (not shown) to increase expression of the epitope recognized by the moAbs. For the competition experiments the compound ND-4043 was incubated for 30min at rt with the cells and not washed away prior to addition of the anti-envelope or the CD4 receptor or coreceptor antibodies. Unlabeled mouse monoclonal anti-CD4 antibodies used included: 13B8 (Beckman Coulter), DB81 [19], Leu3A (BD Biosciences), and 3 separate clones (379, 34924, and 34940) from R&D Systems. Subsequent secondary antibody staining with anti-mouse (Sigma) was performed at 4°C for 15min. Acquisition of data (10000 events/sample) was performed with a Gallios (Beckman Coulter, Inc.) or BD FACS Canto (San Jose, CA) flow cytometer and analyzed by FlowJo software (Tree Star).

### Influenza virus entry assay

A viral entry assay where a β-lactamase protein (Bla) fused to the influenza matrix protein-1 (M1) was packaged as a structural component into influenza virus-like particles (VLPs) has been described previously [20]. Briefly, 293T (NIBSC, UK) and MDCK cells were incubated with the compounds at rt 30min prior to infection with influenza VLPs. Viral entry was then quantified by adding commercially available CCF2-AM substrate (Life Technologies) to the infected cells and detecting substrate cleavage by the presence or absence of the Bla protein. All experiments were performed using a LSRII flow cytometer (BD Biosciences).

### HHV-6 fusion assay

A vaccinia-virus adapted assay was set up to evaluate fusion mediated by HHV-6 [21]. Donor HSB-2 T cells (NIH, USA) were infected with cell-free HHV-6 A (strain GS) and then infected with vTF7-3 expressing the bacteriophage T7 RNA polymerase gene. Many cell types have been shown to be permissive to HHV-6-mediated entry, including HeLa cells. Thus target cells were TZM-bl infected with vCB21R-lacZ, expressing the E. coli lacZ gene linked to the bacteriophage T7 RNA polymerase promoter. The murine anti CD46 moAb J4.48 (Coulter-Immunotech) was used as a control treatment for blockade of HHV-6 fusion.

### Ethics statement

Anonymized samples of peripheral blood were obtained from healthy volunteer donors at the Blood Bank of the San Raffaele Hospital under supervision by the Institutional Review Board of the Ethics Committee of the San Raffaele Hospital in Milan.

## Results

### Inhibitors

The compounds evaluated in the present study were ND-4040, ND-4041, ND-4042, ND-4043, distamycin itself (4044) and its close homolog ND-4045. The chemical structure of the compounds is displayed in Additional file 1: Table S1.

### Inhibition of HIV-1 replication independent of coreceptor usage

**Figure 1:**
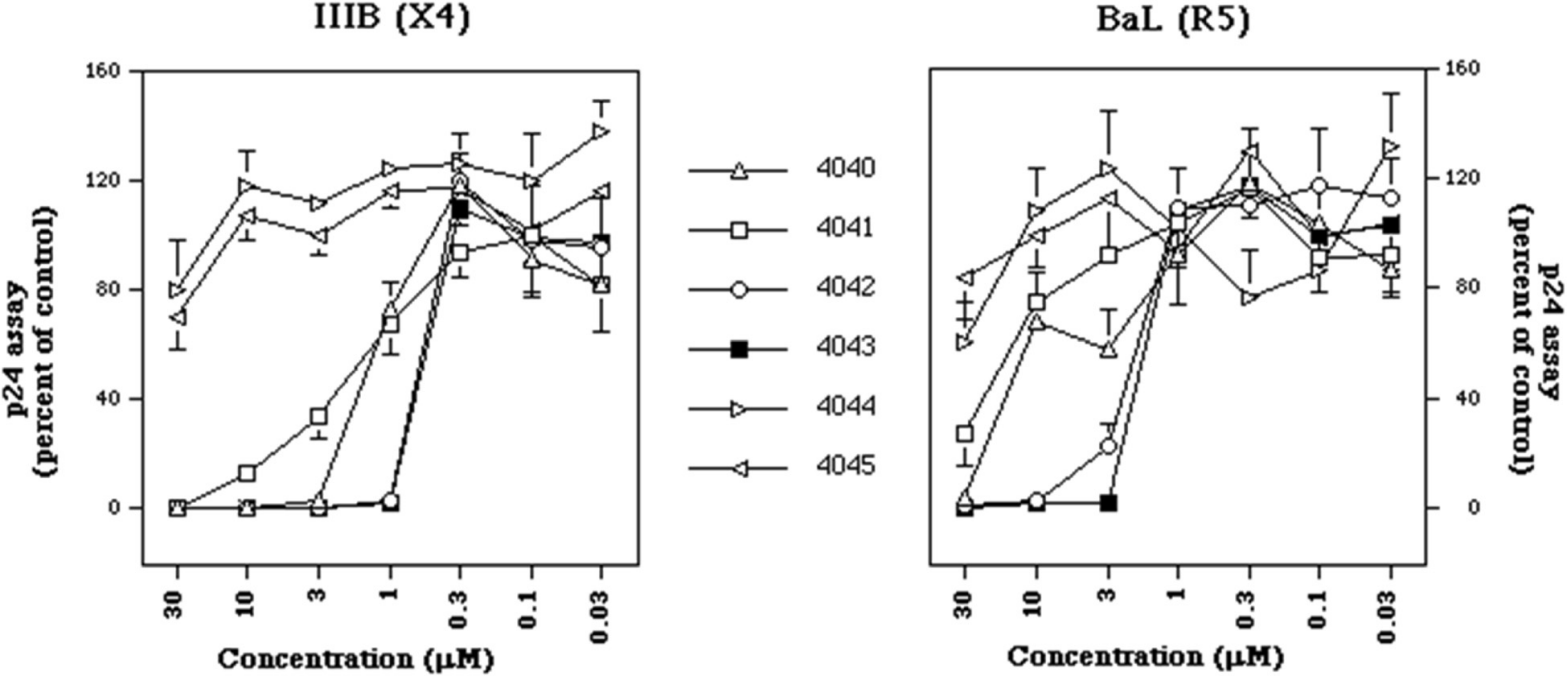
**Effect of the six compounds on HIV-1 replication (left panel X4, right panel R5) measured by the p24 assay on day 7 culture supernatants.** Infections were carried out in triplicate cultures in complete medium plus 10% of heat-inactivated FCS. Data are reported as percent of control which are PBMC in the absence of any compound, but pre-incubated with DMSO at 0.2%, corresponding to the concentration present in 30 μM of the compound. Values refer to mean ± standard deviation (SD) of 4 independent experiments each with triplicate cultures.

The compounds were tested in HIV-1 infection experiments performed with PHA-activated PBMC derived from healthy donors. Two laboratory-adapted strains were used: IIIB, which uses CXCR4 as coreceptor (X4), and BaL, which requires CCR5 as coreceptor (R5) and viral replication was measured by p24 ELISA in day 7 culture supernatants (Figure 1). A wide range of concentrations were evaluated, from 30 μM to 0.03 μM. Control cultures contained a concentration of DMSO equivalent to that of the highest concentration (30 μM) of the compounds. Distamycin (4044) and ND-4045 were not effective, even at the highest concentrations tested, whereas compounds ND-4040, ND4041, ND-4042, ND-4043 were effective against both X4 and R5 HIV-1 viruses at the highest concentrations, but were more powerful against X4 than R5 (Figure 1). Similar results were obtained in day 4 culture supernatants (data not shown). The most active compound was ND-4043, closely followed by ND-4042, then ND-4040 and at last ND-4041. This behavior is summarized in Table 1 which displays the inhibitory concentrations (IC) of the six compounds against X4 and R5 HIV-1. Inhibition of HIV-1 replication by the active compounds also at the highest concentrations was not associated with cytoxicity, as evaluated by the MTT assay (Table 1).

**Table 1:** **Anti-HIV-1 activity of the compounds**

**Compound**	**IC50**	**IC75**	**IC90**	**CC50**
**IIIB (X4)**				
ND-4040	1.6	2.3	3.0	>30
ND-4041	1.9	5.3	12.6	>30
ND-4042	0.7	0.9	1.4	>30
ND-4043	0.6	0.8	1.0	>30
4044	>30	>30	>30	30
ND-4045	>30	>30	>30	28
**BaL (R5)**				
ND-4040	13.7	21.0	27.2	>30
ND-4041	17.8	>30	>30	>30
ND-4042	2.3	3.2	6.6	>30
ND-4043	2.0	2.6	3.0	>30
4044	>30	>30	>30	25
ND-4045	>30	>30	>30	30

Inhibitory (50, 75 and 90%) and cytotoxic (50%) concentrations (μM) of the six compounds against X4 and R5 HIV-1 viruses in infected PBMC.

**Figure 2:**
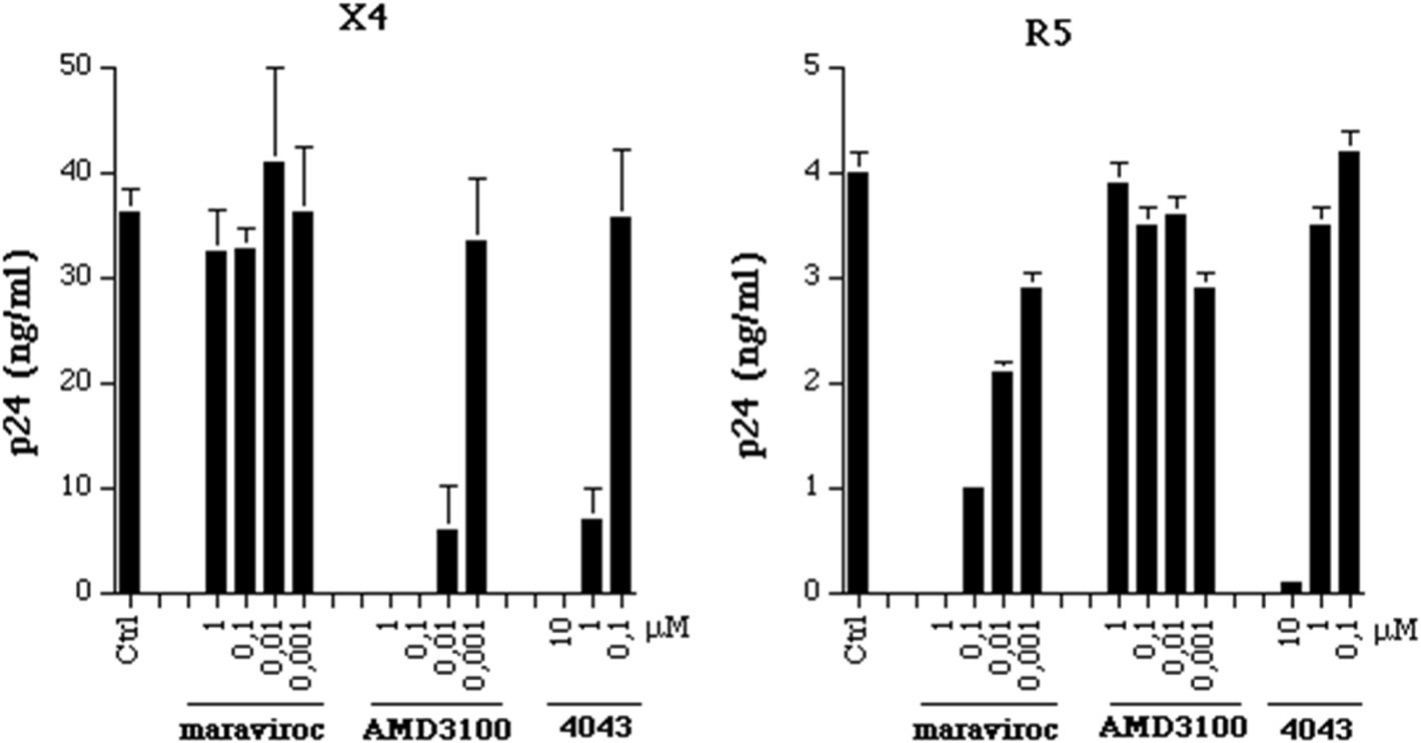
**Comparison of maraviroc, AMD3100 and ND-4043 on inhibition of HIV-1 replication (left panel X4, right panel R5).** The antigen p24, expressed as ng/ml, is measured at day 7 post infection and data are reported as mean ± SD of three replicates. Infections were carried out in complete medium plus 10% of heat-inactivated FCS. Control PBMC were incubated with DMSO at 0.2% corresponding to the concentration present in 30 μM of ND-4043. The tested concentrations of compounds (ten fold dilutions) are shown in the figure.

The activity of ND-4043 was compared to that of two reference small molecules with selective inhibition on R5 or X4 replication, maraviroc and AMD3100, respectively (Figure 2). As expected, maraviroc and AMD3100 had no effect on X4 and R5 HIV-1 replication, respectively, at all tested concentration. AMD3100 inhibited X4 HIV-1 replication in a concentration-dependent manner with complete inhibition up to 0.01 μM, whereas maraviroc displayed a complete blockade of R5 HIV-1 replication at 0.1 μM. ND-4043 inhibited both X4 and R5 HIV-1 replication at 1 μM and 10 μM, respectively.

### Studies on the mechanism of antiviral activity

We first evaluated the activity of these compounds at HIV-1 transcriptional and post-transcriptional level employing HIV-1 chronically infected cell lines, namely ACH-2 [22] and U1 [23], which offer a high-throughput model system for evaluating inhibition with good standardization of the results. Treatment of these cell lines with TNF-α leads to HIV-1 transcription, through activation of the transcription factor NF-κB [22], translation and secretion of virions in culture supernatants. The compounds did not inhibit TNF-α-induced HIV-1 expression at any concentration in both cell lines (data not shown). It was concluded that these compounds were not acting at the transcriptional and post-transcriptional level of HIV-1 replication. The latter was important to investigate since suramin itself was reported to inhibit the reverse–transcriptase of oncogenic retroviruses [24].

**Figure 3:**
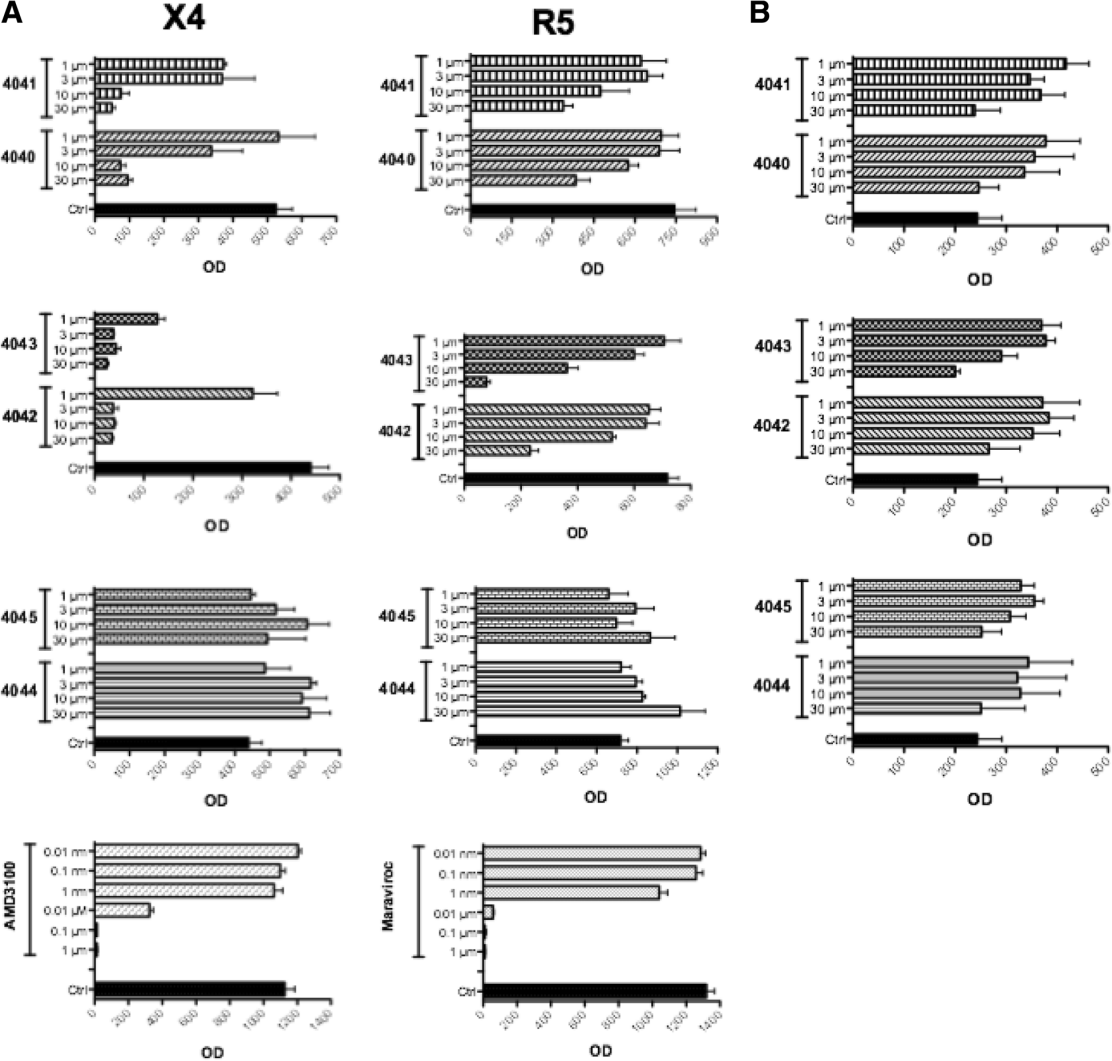
**Effect of different concentrations of the six compounds plus AMD3100 and maraviroc on HIV-1-mediated fusion (A) and cytotoxicity (B).** Fusion assays were carried out in complete medium plus 2.5% of heat-inactivated FCS with SupT1-IIIB (X4) or PM1-BaL (R5) as donor cells and TZM-bl as target cells (left panel X4, right panel R5). Viral fusion data, expressed as OD, are presented as mean ± SD of three replicates on the x axes, whereas concentrations of tested compounds are reported on the y axes. **B**. MTT assay on day 4 cultures of TZM-bl not infected with vaccinia virus, in the presence or absence of different concentrations of the six compounds. Data are expressed as OD and represent mean ± SD of three replicates. Control TZM-bl were incubated with DMSO at 0.2% corresponding to the concentration present in 30 μM of the compounds.

Subsequent experiments were performed to evaluate the earliest stages of the HIV-1 life cycle, namely fusion and entry. The compounds were tested in a modified version of a previously established recombinant vaccinia virus-based assay [25] that measures activation of a reporter gene upon fusion of two distinct cell populations. The T-cell lines SupT1 and PM1 chronically infected with HIV-1 IIIB (X4) and BaL (R5), respectively, were used as effector cells, whereas TZM-bl were used as target cells (Figure 3A). As expected, compounds 4044 and ND-4045 did not affect R5- and X4-mediated fusion also at the maximum concentration used, whereas the other four compounds were active. Entry of the X4 virus (left panel) was inhibited by all four compounds at 30 and 10 μM; ND-4042 and ND-4043 were effective at 3 μM, but only ND-4043 still exerted 65% inhibition at 1 μM. Entry of the R5 virus (right panel) was less efficiently counteracted, in line with the data obtained with viral replication. The inhibitory activity of ND-4043 on R5 HIV-1-induced fusion was complete at 30 μM and about 50% at 10 μM. The control molecules AMD3100 and maraviroc selectively blocked entry of X4 and R5 HIV-1, respectively, in a concentration-dependent manner. Plain TZM-bl cells (not infected with vaccinia virus to express the reporter gene) were cultured in the absence and presence of different concentrations of the six compounds for four days and cytotoxicity was evaluated by the MTT assay (Figure 3B). No substantial inhibitory effect was reported even at the highest concentrations used, underlining the importance of fusion inhibition.

Of note, inhibition by these compounds of R5 HIV-1 appears less potent in the fusion compared to the PBMC assay; this is most likely due to CCR5 levels which are more elevated in TZM-bl cells than in primary PBMCs, resulting in more efficient fusion kinetics.

**Figure 4:**
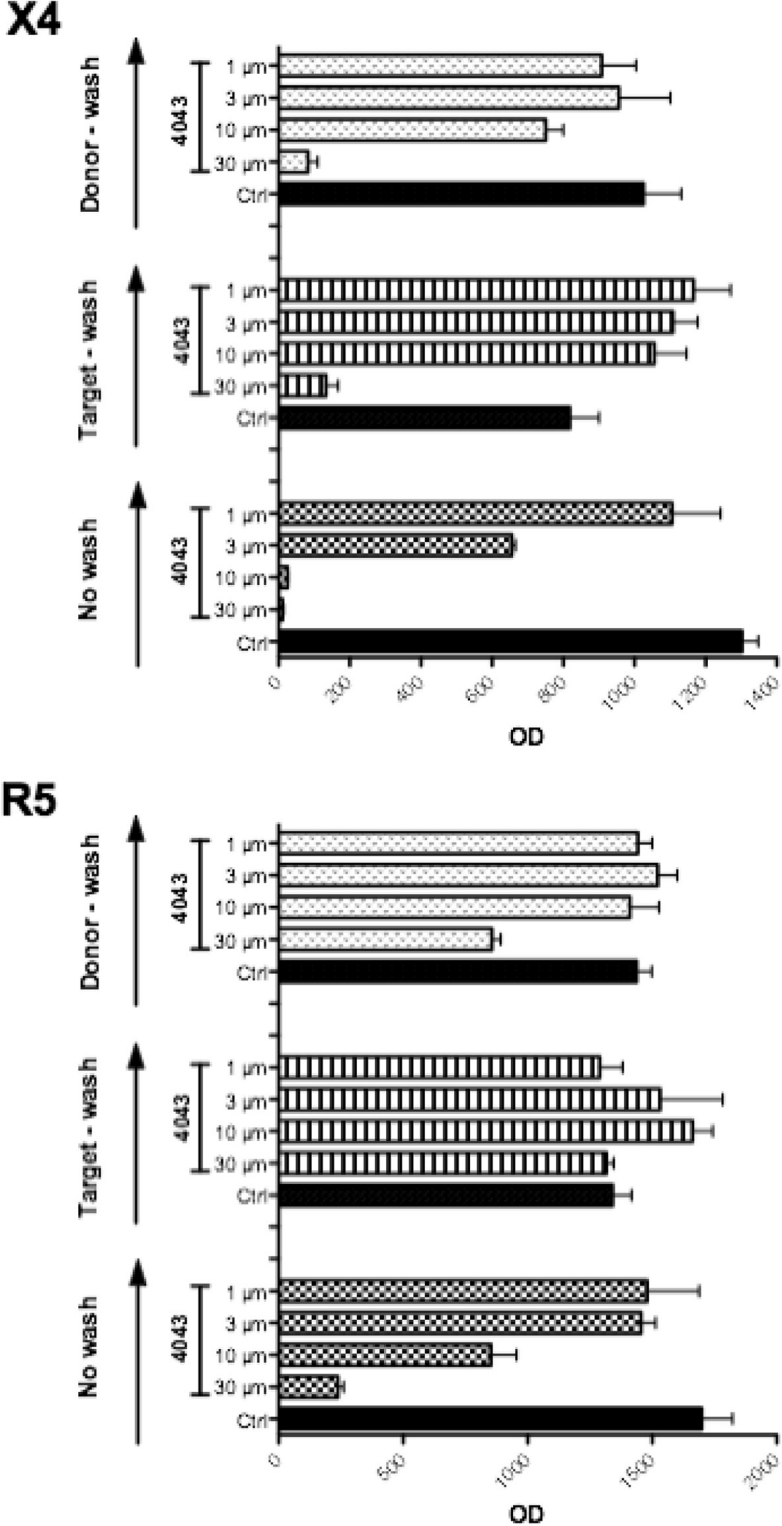
**Differential effect of ND-4043 on donors and targets in HIV-1-mediated fusion (upper panel X4, lower panel R5) entry.** The fusion assay was carried out in complete medium plus 2.5% of heat-inactivated FCS. Both donor and target were 3T3 cells: as donors they were infected with vaccinia virus expressing X4 (vCB41) or R5 (vCB43) HIV-1 envelope, as targets they were stably transfected with CD4 plus CXCR4/CCR5. Ctrl = control is the mixture of the donor and target populations of 3T3 cells in the absence of ND-4043. Three incubation conditions were compared in the same experiment. Viral fusion is expressed as OD and reported data are mean ± SD of three replicates. The compound ND-4043 is tested at 30, 10, 3 and 1 μM.

A deeper investigation of the mechanism of action at molecular level was carried out with compound ND-4043 (Figure 4). A 3T3-based fusion assay was employed in which 3T3 equipped with appropriate HIV-1 entry molecules acted as both effector and target cells. This is a rigorous experimental model since effector 3T3 cells do not express endogenous CCR5 or CXCR4, the latter being expressed endogenously in several human cell lines, including PM1 and SupT1. The fusion assay was carried out with three different modalities (Figure 4): as per protocol, i.e. ND-4043 was added to the co-cultured 3T3 populations and left in the wells throughout the culture period without washing (“no wash”, bottom panels of X4 and R5 data sets); ND-4043 was incubated for 30 min only with the donor or target cells, removed by washing, and then donor and target cells were co-cultured (“donor–wash” and “target–wash”, respectively). As expected, inhibition of X4 and R5 HIV-1 entry by ND-4043 was confirmed in the no-wash modality, with the X4 envelope-mediated fusion better inhibited than the R5 counterpart. In the donor-wash condition, inhibition of X4 and R5 was reduced, but maintained, whereas in the target–wash condition, X4 inhibition was maintained but R5 inhibition was lost. These results indicate that inhibition of viral entry by ND-4043 involves the donor more than the target cell, underlying a potential interaction with the viral envelope and, to a lesser extent, with the cell receptor complex formed by CD4 and CXCR4.

### Mapping of the HIV-1 envelope region targeted by ND-4043

**Figure 5:**
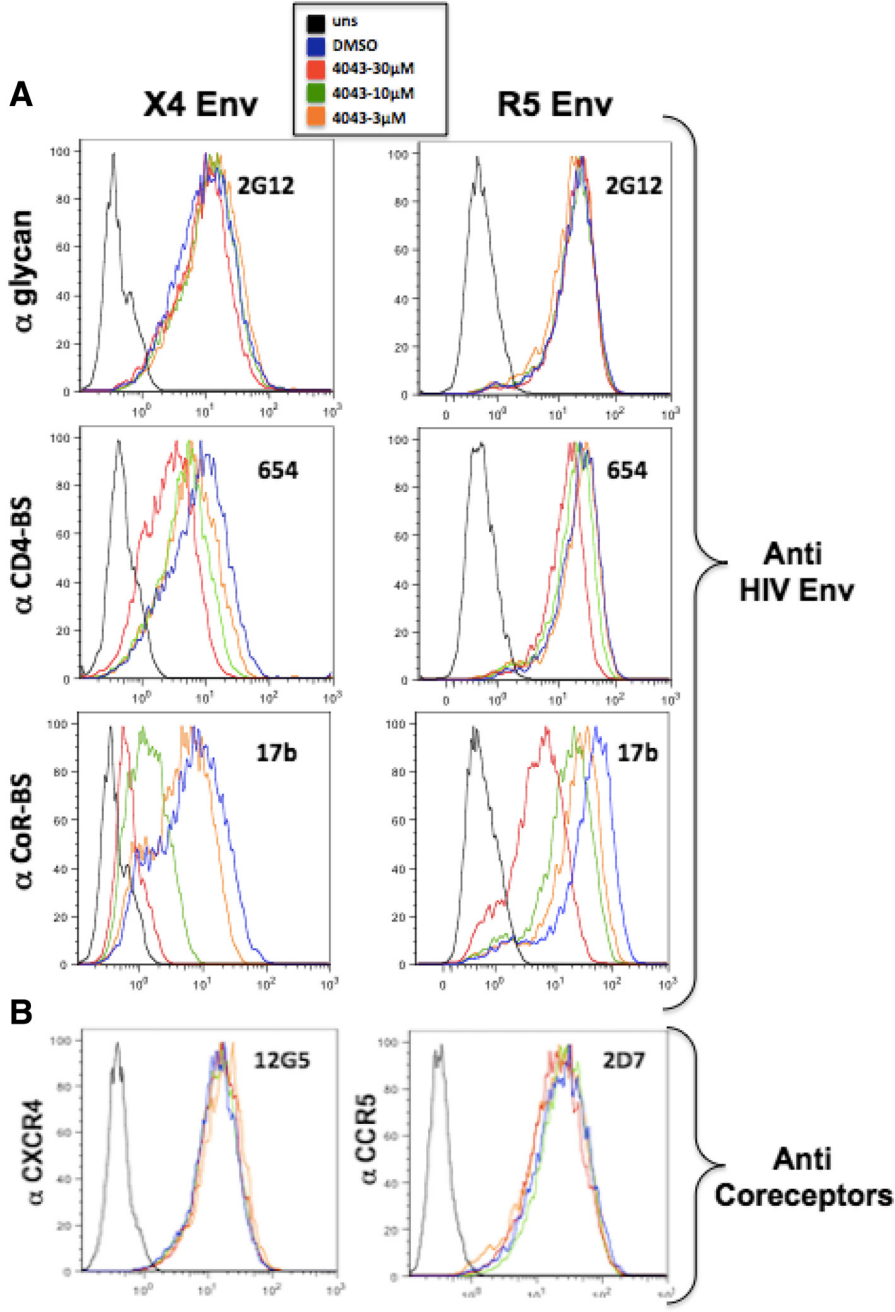
**Flow cytometric analyses in 3T3 cells expressing Env (A) or CXCR4 and CCR5 (B).** Three concentrations (30, 10 and 3 μM) of the ND-4043 compound were used to compete with binding of anti HIV envelope **(A)** or anti coreceptor **(B)** moAbs. Incubation with ND-4043 was carried out in PBS plus 2% of heat-inactivated FCS. DMSO refers to control cells stained with the moAbs but pre-incubated only with DMSO at 0.2%, corresponding to the concentration present in 30 μM of the compound; uns: unstained cells. A color-coded legend is displayed within the figure.

We took advantage of the availability of several human moAbs directed against different epitopes of gp120 [26] to perform flow cytometry experiments aimed at the characterization of the envelope region targeted by ND-4043. 3T3 cells infected with vaccinia virus constructs expressing either X4 (left panel) or R5 (right panel) envelope were incubated with ND-4043 at three concentrations prior to addition of the human anti-envelope moAbs (Figure 5A). The broadly neutralizing 2G12 moAb recognizes a unique mannose-dependent epitope on gp120 that is not directly associated with the receptor-binding sites on this protein [27]. Neither binding of the anti glycan 2G12 moAb (Figure 5A, upper panels) nor the binding of the anti gp41 2F5 moAb (data not shown) were affected. Substantial competition with the anti CD4-binding site (CD4-BS) moAb 654 [28] was observed with the X4 and, to a lesser extent, with the R5 envelope expressing cells (Figure 5A, middle panels). Similar results were obtained with another anti CD4-BS moAb, b12 [29] (data not shown). Of interest, the anti coreceptor-binding site (CoR-BS) moAb 17b [30] was displaced strongly and in a concentration-dependent manner by ND-4043 on both envelopes (Figure 5A, lower panels). Similar results were also obtained with another anti CoR-BS moAb, 48d [30] (data not shown). Moreover, 3T3 cells expressing CXCR4 (left panel) or CCR5 (right panel), but not HIV-1 envelope, were stained with anti CXCR4 and anti CCR5 moAbs (Figure 5B). No competition with anti coreceptor moAb binding was achieved by prior ND-4043 incubation, suggesting that ND-4043 specifically targets the CoR-BS on the HIV-1 envelope, but not directly the coreceptors.

**Figure 6:**
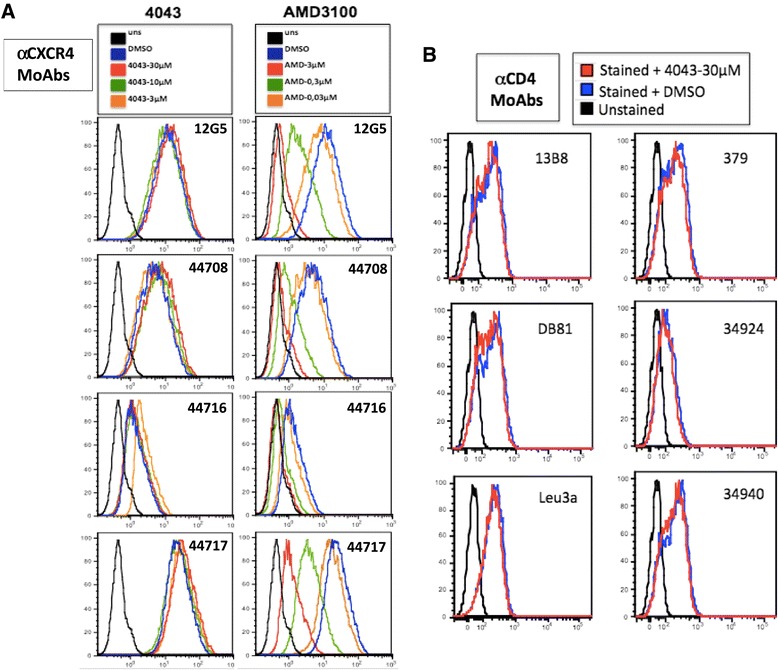
**Flow cytometric analyses in uninfected PM1 cells.** ND-4043 is used to compete with binding of 4 anti CXCR4 **(A)** and 6 anti CD4 **(B)** moAbs. Incubation with ND-4043 was carried out in PBS plus 2% **(A)** or 1% **(B)** of heat-inactivated FCS. **A**. ND-4043 was used at three concentrations (30, 10 and 3 μM) and also AMD3100 (3, 0.3 and 0.03 μM). **B**. ND-4043 was used at 30 μM. DMSO refers to control cells stained with the moAbs but pre-incubated only with DMSO at 0.2%, corresponding to the concentration present in 30 μM of the compound; uns: unstained cells. A color coded legend is displayed within the figure.

Since our earlier experiment (Figure 4) showed that inhibition of X4, but not R5, fusion was still achieved when ND-4043 was incubated only with target cells, further analyses were performed to evaluate competition by ND-4043 towards binding of a panel of moAbs directed against CXCR4 (Figure 6A). Treatment of PM1 cells with ND-4043 did not affect binding of four anti-CXCR4 moAbs, including the one used by Howard et al. [12] in the study with a congener of this class of molecules. AMD3100 instead inhibited binding of the four moAbs in a concentration-dependent manner. Similar results were obtained also with HOS-CXCR4 expressing cells (data not shown). Finally, ND-4043 at the highest concentration did not inhibit binding of six different anti CD4 moAbs in PM1 cells (Figure 6B). Taken together, these data suggest that ND-4043 does not bind to CXCR4 or CD4 on target cells.

### Sensitivity of different viruses to entry inhibition

**Figure 7:**
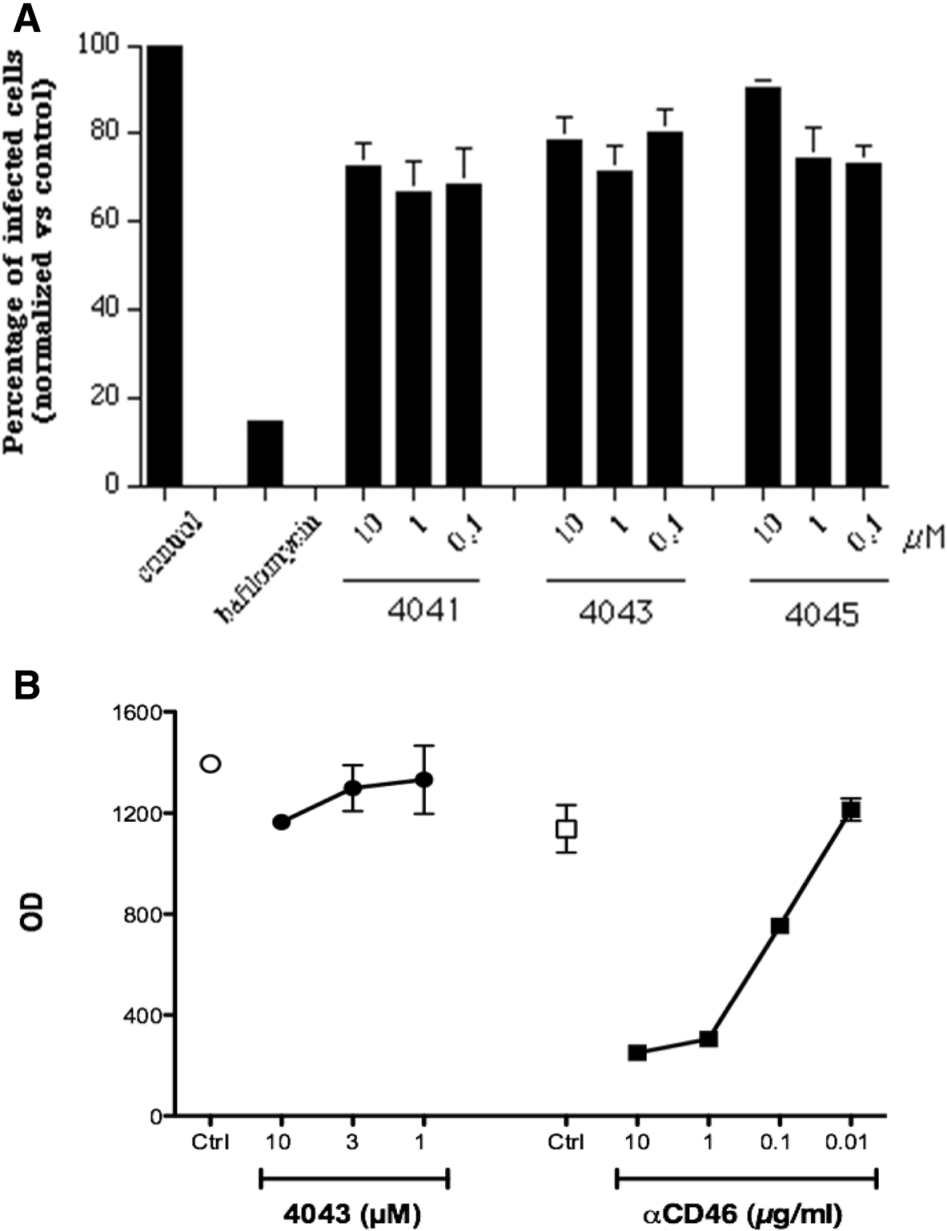
**Effect of compounds on entry of different viruses. A**. influenza virus (flu) VLP entry in MDCK cells. ND-4041, ND-4043 and ND-4045 were tested at three concentrations; bafilomycin (100 nM) was used as positive control of flu entry inhibition. Data are expressed as percentage of control (cells in the absence of compounds) and represent mean ± SD of three replicates of two independent experiments. **B**. HHV-6 virus-mediated fusion of HSB-2 and TZM-bl cells. Viral entry is expressed as OD and data are mean ± SD of three replicates. ND-4043 was tested at three concentrations and the anti CD46 moAb (10, 1, 0.1 and 0.01μg/ml) was used as positive control of entry inhibition. Data are representative of two independent experiments.

Given the inhibition of HIV-1 fusion by the active compounds, we sought to understand whether their mechanism of action was specific to HIV entry. Influenza viruses infect target cells using a surface glycoprotein, hemagglutinin, which has fusion and trimerization properties that resemble those of gp160, but binds to a different receptor (sialic acid). Entry of influenza A virus was therefore evaluated by a virus-like particle (VLP)-based enzymatic assay, with flow cytometry read-out set up for sensitive detection of viral entry [20]. Entry of influenza VLPs into canine kidney cells was evaluated (Figure 7A). Pretreatment of cells with bafilomycin A1, a chemical inhibitor of endosomal acidification, inhibited viral entry significantly in canine kidney cells. This effect was specific to VLP entry, and not due to inhibition of beta-lactamase substrate entry, as non-infected cells with or without bafilomycin A1 had similar levels of CCF2AM substrate present in the cytoplasm after its addition (not shown). The dimeric compound ND-4043, its monomer ND-4041 and the HIV-1 inactive compound ND-4045, used as control, showed none or negligible dose-response effects on influenza A virus entry at any of the tested concentrations. Similar results were obtained with human embryonic kidney 293 cells (data not shown).

The DNA human herpes virus-6 (HHV-6) binds to CD46 [21], an ubiquitous immunoregulatory receptor very different from the receptor for HIV. Thus, fusion induced by this herpes virus was also evaluated in the presence and absence of ND-4043 (Figure 7B). Fusion occured when the T-cell line HSB-2, infected with the HHV-6 A GS strain, is mixed with the TZM-bl cell line (Figure 7B), while no fusion occured with uninfected HSB-2 cells (data not shown). ND-4043 showed negligible inhibition of HHV-6-induced fusion at any of the tested concentrations, whereas an anti CD46 moAb inhibited fusion in a concentration-dependent manner.

### Breadth of HIV-1 inhibitory activity

**Figure 8:**
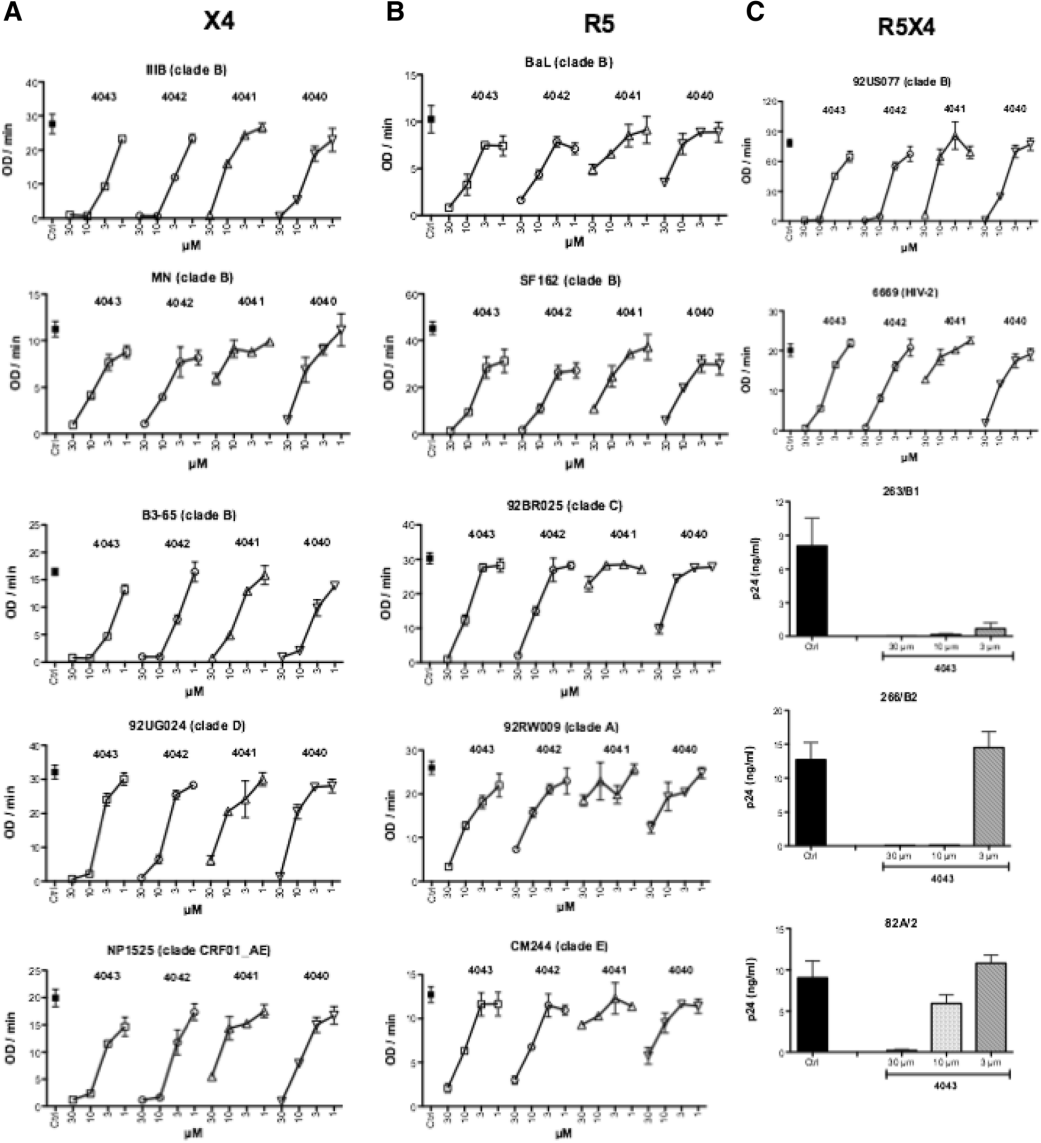
**Breadth of anti-HIV activity by the active compounds.** Fusion assays were performed with target TZM-bl cells plus donor PM1 cells chronically infected with 5 X4 **(A)**, 5 R5 **(B)** and 2 R5X4 viruses **(C)**. Each panel represents a single experiment carried out with three replicates. Fusion assays were performed in complete medium plus 2.5% of heat-inactivated FCS. The four active compounds have been tested at the indicated concentrations. Viral entry is expressed as OD/min and data are reported as mean ± SD of the three replicates. **C**. The p24 assay, expressed as ng/ml, was performed with day 7 supernatants from PBMC infected with the three indicated R5X4 viruses and only ND-4043 was tested (30, 10, 3 μM). Values refer to mean ± SD of three replicates.

The breadth of the HIV-1 inhibitory activity exerted by the four active compounds was investigated (Figure 8). To this aim, fusion assays were performed with PM1 cells chronically infected with primary strains of HIV-1 of different clade and tropism as envelope donor cells and the TZM-bl as target cells expressing host cell receptors CD4, CXCR4 and CCR5. Four concentrations of each compound were tested on entry involving 5 X4 (Figure 8A), 5 R5 (Figure 8B) HIV-1 strains, including the prototypic IIIB (X4) and BaL (R5). In agreement with the data reported in the present paper, fusion mediated by X4 envelopes was more efficiently inhibited than the one mediated by R5 envelopes. In both cases inhibition was concentration-dependent. The dimeric compounds ND-4043 and ND-4042 were the most active inhibitors, followed by ND-4040 and ND-4041, which acted poorly on R5 viruses of clade A, C and E.

Finally, dualtropic R5X4 viruses were also tested for sensitivity to inhibition (Figure 8C). ND-4043 and ND-4042 were comparable in their inhibitory effect, while ND-4040 and especially ND-4041 were less active in the cell fusion assay (Figure 8C, two upper panels). ND-4043 induced a concentration-dependent inhibition of viral replication of additional three primary R5X4 strains in the PBMC infection assay (Figure 8C, three lower panels).

## Discussion

Dystamicin (4044) and the closely related ND-4045 displayed some cytotoxicity and irrelevant HIV inhibitory effect at the concentrations tested *in vitro*. Conversely, four novel compounds, ND-4040, ND-4041, ND-4042, ND-4043, exerted inhibition of viral replication in the low micromolar range with no evident cytotoxicity.

Noteworthy, both X4 and R5 strains of HIV-1 were inhibited, and X4 consistently more effectively than R5. Experiments assaying the early stages of the viral life cycle, namely fusion, were consistent with a mechanism of action of these inhibitory compounds at the level of viral entry. This is in agreement with a study on a previous compound of the same class (NSC 651016), which reported an anti-HIV effect at viral entry [12]. Herein ND-4043 competes with binding of certain anti HIV envelope, but not with binding of anti coreceptor moAbs. This is a novel finding and differs from earlier data reporting NSC 651016 to target CCR5 and CXCR4 [12]. Of interest, suramin itself was shown to target a multibranched V3 peptide of gp120, inhibiting binding of HIV-1 envelope to galactosylceramide, the receptor for HIV-1 in human colon epithelial cells [31].

HIV entry is a multi-step process requiring several players and a coordinated sequence of events: binding of gp120 to the primary receptor CD4, a conformational change of gp120 leading to binding of the CoR-BS of gp120 to the CCR5 or CXCR4 coreceptor, unmasking of gp41 and its insertion into the cell membrane leading to fusion of the viral and cell membrane and, finally, entry of the HIV-1 core into the cell [32,33]. We provide evidence that ND-4043 targets the CD4-BS and the CoR-BS of gp120 to precisely counteract the initial conformational changes within the HIV envelope that support successful viral entry.

It is intriguing how the mechanism of action of ND-4043 closely resembles that of the human alpha defensin-5 (HD-5) [34], a peptide of the innate anti-microbial host defense response, which binds both the CD4-BS and the CoR-BS of gp120. The HIV inhibitory effect of HD-5 is lost or reduced in the presence of bovine serum in culture. We also observed that inhibition by a suboptimal concentration of ND-4043 is greatly enhanced (about 75%) when the HIV fusion assay is performed in the absence of FCS compared to the typical 2.5% concentration of FCS (data not shown). Nevertheless, binding of serum proteins by NSC 651016, a congener of the suradista class of molecules, may account for its remarkable stability and long half-life in plasma, as documented by pharmacokinetic studies in mice [11] and humans [10].

The current ART armamentarium encompasses two drugs that act at the entry level, enfuvirtide and maraviroc. Enfuvirtide [35], blocks gp41-mediated fusion, the last step of the HIV entry sequence of events, irrelevant of viral tropism. However, being a 36-AA polypeptide, it needs to be delivered twice daily by subcutaneous injection to obtain therapeutic concentrations, a modality that hampers patients’ compliance. Maraviroc [36,37] is orally bioavailable and, by targeting the CCR5 coreceptor, is suitable for treatment of patients harboring R5 viruses. R5 infections are the majority, but a substantial number of patients harbor dualtropic viruses (R5X4) [38-42]. Thus, the unique feature of ND-4043 and congeners to inhibit R5, X4 and R5X4 HIV-1 viruses identifies these compounds as potentially broad-spectrum treatments. Pure X4 viruses are rare (around 1-3%), but R5X4 viruses have been found in about 15-20% of treatment-naïve individuals [38,39] and in 40-48% of treated and multiexperienced patients [39-42]. An association between non-R5 virus infection and more rapid CD4 T-cell loss both in treated and ART-naive subjects has been reported [39,43,44]. Importantly, combination ART including maraviroc requires coreceptor usage screening [45] of patients’ virus to exclude those who harbor R5X4 or X4 viruses. Indeed maraviroc was shown to be neither superior nor noninferior to placebo in treatment-experienced patients with non-R5 HIV-1 [46]. Thus the availability of another drug targeting viral entry, rather than enfuvirtide, provides a desirable treatment option for a relatively large proportion of individuals with non-R5 HIV-1 viruses, who are excluded by current maraviroc-containing ART. Future in *vitro* investigation is required to evaluate the inhibitory effect of these compounds tested together with components of the antiretroviral combination therapy, including NNRTI/NRTI, PI, integrase inhibitors, or investigational compounds currently in clinical trials such as the attachment inhibitor BMS-378806 [47].

The unique pharmacokinetic profile displayed by members of this class of compounds, with terminal plasma half-life in humans in the order of one month, represents a substantial treatment advantage for patients, with a significant impact on compliance and, consequently, efficacy. In many instances, pharmacokinetic/pharmacodynamic properties may represent major determinants in the exploitability of certain compounds, rather than potency by itself [48]. Indeed a novel and currently pursued strategy is to provide long-acting formulations of antiretrovirals [49], as monthly or bi-monthly injections may provide a safer and more efficacious treatment option to oral dosage.

Another context in which this class of compounds could be important is vertical mother-to-child transmission, a significant means of HIV-1 transmission in developing countries. The efficacy of anti-HIV therapy in blocking vertical transmission during the perinatal phase has been documented [50]. However, economic and logistic problems limit the use of programs for prevention of vertical transmission. In this context, a long-acting drug with the potential of twice monthly dosing may represent an enormous medical advantage.

Discovery and optimization of CXCR4 antagonists as anti-HIV therapeutics has been attempted; however, the selective X4 entry inhibitor, AMD3100, has been licensed and marketed as a hemapoietic stem cell mobilizer (Plerixafor) [51]. Of interest, in the side-by-side comparison performed in this study AMD3100 and maraviroc were approximately 100 fold more active than ND-4043 on X4 and R5 inhibition, respectively. Nevertheless, co-administration of two separate drugs would be required to exert the broad anti-HIV effects mediated by ND-4043 alone. Moreover, virologic failure to maraviroc was associated with emergence of CXCR4-using virus in 57% of subjects in whom a further tropism test was obtained at the failure time-point [52]. In the case of ND-4043, which exhibits more potent inhibition against CXCR4-utilizing viruses, the hypothetical risk of directing R5X4 strains towards use of CCR5 would result in the positive outcome of making those viruses more susceptible to inhibition by maraviroc. A therapeutic strategy that lately is being proposed is to combine CD4 mimics [53], inhibitors of the CD4-gp120 interaction, together with anti coreceptor molecules, or making hybrids [54]. This strategy appears to be recapitulated by the action of ND-4043 alone which competes for binding of anti CD4-BS and, to a greater extent, of anti CoR-BS moAbs on HIV-1 envelope.

## Conclusions

This study identifies novel inhibitors of HIV-1 entry capable of inhibiting both X4 and R5 viruses. Mechanistic studies with ND-4043, the most active compound, indicate that inhibition of viral entry involves the CD4-BS and the CoR-BS on HIV Env. This inhibitor may represent an important component of combination therapy in patients with viral resistance to current entry inhibitors and prove particularly advantageous to those who harbour dualtropic R5X4 viruses because of its distinct mechanism of action. Selectivity of inhibition of HIV entry is highlighted by the irrelevant effect exerted by ND-4043 on assays addressing entry of influenza and HHV-6 viruses, both of which use very different cellular receptors.

ND-4043 is expected to share the favorable pharmacokinetics and low cytotoxicity displayed by a congener of the same class that has been used in a phase I clinical trial as an anti-angiogenic agent. Thus, further investigation is warranted aiming towards development of ND-4043, or other members of the same class, as potential anti-HIV-1 drugs.

## Additional file

Additional file 1: Table S1. Chemical structure of the compounds used in this study.

## Abbreviations

PHA: Phytoemagglutinin
PBMC: Peripheral blood mononuclear cells
RT: Reverse transcriptase
rt: room temperature
Bla: β-lactamase protein
OD: Optical density
SD: Standard deviation
NRTI: Nucleoside reverse transcriptase inhibitors
NNRTI: Non-nucleoside reverse transcriptase inhibitors
PI: Protease inhibitors
