# Supplementary figures and images for: Characterization of HIV-1 entry inhibitors with broad activity against R5 and X4 viral strains
Source: J Transl Med. 2015 Apr 2;13:107. doi: 10.1186/s12967-015-0461-9 (PMC4399250; doi:10.1186/s12967-015-0461-9)

**Table 1.** Chemical structure of the compounds used in this study.
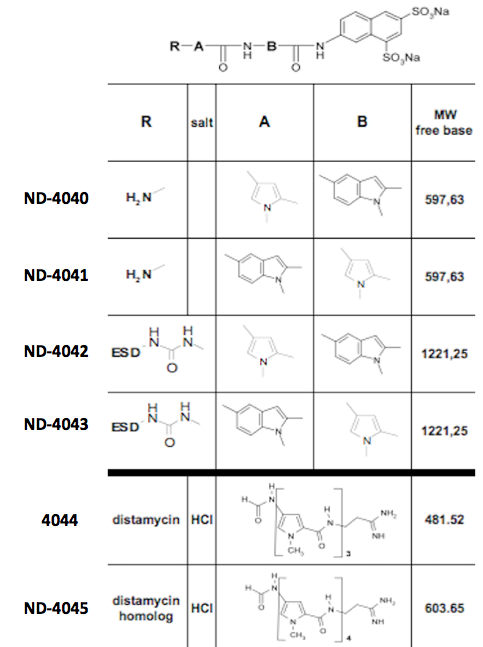

Supplement: Additional file 1: Table S1. — Chemical structure of the compounds used in this study. [file 12967_2015_461_MOESM1_ESM.doc]
